# Assessing the Accuracy, Completeness, and Reference Quality of GPT-4 and Google for Gynecologic Cancer Information: Comparative Quantitative Study

**DOI:** 10.2196/76471

**Published:** 2026-09-29

**Authors:** Alexandra H Smick, Martins Ayoola, Rajeshree Rajpara, Isabel Lazo, Dana M Chase

**Affiliations:** 1 Gynecologic Oncology University of California, Los Angeles Los Angeles, CA United States; 2 Undergraduate College University of California, Los Angeles Los Angeles, CA United States; 3 Gynecologic Oncology Olive View-UCLA Medical Center Sylmar, CA United States

**Keywords:** artificial intelligence, AI, gynecologic cancer, patient education as topic, large language models, gynecologic neoplasms, health literacy, cancer communication, patient information materials

## Abstract

**Background:**

Patients with newly diagnosed gynecologic cancers often seek information online, but the quality of available resources may be inconsistent. Although GPT-4 may offer an alternative to traditional internet search engines, its performance remains largely under-studied in gynecologic oncology.

**Objective:**

This study aimed to compare the completeness, accuracy, and reference quality of responses generated by GPT-4 with those generated by Google in clinical scenarios involving a new diagnosis of a gynecologic cancer.

**Methods:**

Clinical scenarios representing early- and advanced-stage endometrial, ovarian, and cervical cancers were developed by gynecologic oncologists using publicly available patient education materials. Each scenario included 4 standardized questions addressing etiology, prognosis, treatment, and treatment efficacy. GPT-4 and Google were queried for each question, with new sessions for GPT-4 and private browsing for Google to minimize bias. Responses were independently evaluated by 4 gynecologic oncology experts who were blinded to each other’s ratings. Accuracy was scored on a 6-point Likert scale; completeness and reference quality were scored on 3-point scales. Reference quality was categorized as low (commercial), medium (institutional or government), or high (peer reviewed). Optional free-text reviewer comments were collected and summarized descriptively to provide context for the quantitative findings. Descriptive statistics and Wilcoxon signed-rank tests were used for analysis.

**Results:**

Across 6 clinical scenarios and 21 standardized questions (N=84 total responses), GPT-4 outperformed Google across all evaluated domains. The median accuracy score was 6.00 (IQR 5.00-6.00) for GPT-4 and 5.00 (IQR 4.00-6.00) for Google (*P*=.04). The median completeness score was 3.00 (IQR 3.00-3.00) for GPT-4 and 2.00 (IQR 1.00-3.00) for Google (*P*=.009). Reference quality was also higher for GPT-4, with a median score of 3.00 (IQR 3.00-3.00) compared to 2.00 (IQR 2.00-2.00) for Google (*P*=.009). Reviewer comments noted that GPT-4 provided more accurate, comprehensive, and personalized responses, while Google returned less detailed content from general consumer health websites rather than peer-reviewed sources.

**Conclusions:**

GPT-4 may serve as a reliable and high-quality resource for information in gynecologic oncology. Compared to Google, it delivered more accurate and complete content, with higher-quality references. Further research is needed to assess the readability and accessibility of GPT-4–generated content across diverse patient populations.

## Introduction

Approximately 100,000 individuals are diagnosed with gynecologic malignancy each year in the United States [1]. Following a new cancer diagnosis, patients frequently seek supplemental educational materials to better understand their condition and treatment options. Traditional resources, such as those from professional societies and academic institutions, are often comprehensive but presented in generalized formats, requiring patients to interpret the content in the context of their own diagnosis and clinical stage [2].

AI, particularly large language models (LLMs) such as GPT-4, has gained significant attention in medicine due to its ability to generate rapid, detailed responses based on vast amounts of textual data. ChatGPT, developed by OpenAI, is powered by the GPT-4 language model and accessible to the public through the ChatGPT Plus subscription. In contrast, Google is a search engine that retrieves existing content from across the internet using proprietary algorithms [3,4]. As of mid-2024, Google began integrating its own LLM, Gemini, into certain aspects of its platform. However, the standard search interface continues to primarily direct users to external web pages rather than generating original text. Previous research has explored the utility of LLMs for clinical tasks such as staging lung cancer, summarizing histopathology reports, and supporting diagnostic decision-making [5,6]. Although early findings are promising, concerns remain regarding the accuracy, reliability, and lack of citation transparency of AI-generated content, reinforcing the need for expert oversight.

As GPT-4 becomes increasingly accessible to the public, patients are likely to turn to it, alongside traditional internet search engines, for information about their diagnosis [2]. Prior studies in oncology have evaluated the quality and readability of GPT-4’s responses to patient questions, highlighting variability in content accuracy and source quality [7,8]. Other investigations have emphasized the potential of AI to improve health literacy by simplifying medical content, yet its role in reliably supporting patient education remains unknown [9]. Within gynecologic oncology specifically, research on AI-based educational tools is limited. Early studies have examined GPT-4’s ability to provide guideline-concordant recommendations for ovarian cancer diagnosis and management, but no prior work has systematically evaluated its performance in delivering high-quality informational content to newly diagnosed patients across multiple gynecologic cancers [10,11].

This study aimed to assess GPT-4’s effectiveness in generating patient-directed informational content in the context of newly diagnosed gynecologic cancers. We compared GPT-4 responses with Google search results across standardized clinical scenarios, focusing on 3 key domains: accuracy, completeness, and reference quality. Our findings contribute to the growing body of literature evaluating AI’s role in digital health and its potential integration into patient-centered cancer education.

## Methods

### Study Design

This was a comparative, cross-sectional evaluation of an AI-based LLM (GPT-4; OpenAI) and a standardized internet search engine (Google; Alphabet Inc) using predefined gynecologic oncology clinical scenarios and patient-centered questions (Figure 1). Methods were structured and reported in accordance with the CHART (Chatbot Health Advice Reporting Tool) guideline for chatbot health advice studies [12].

**Figure 1:**
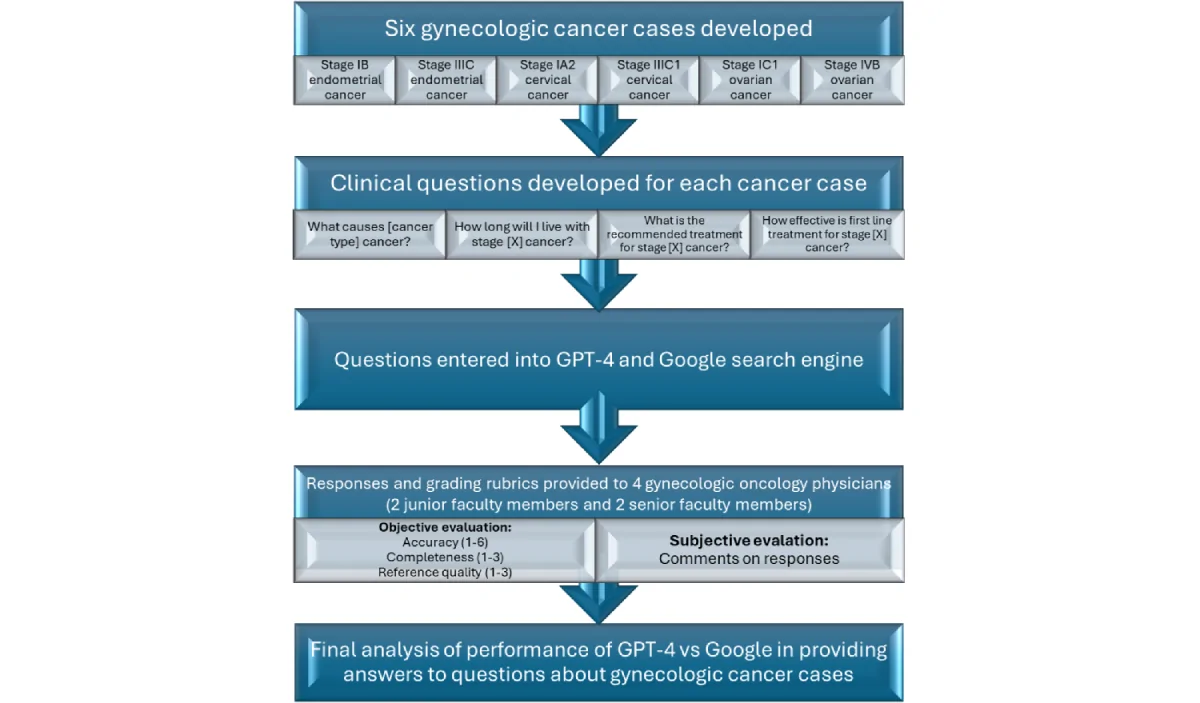
Overview of the study design and evaluation process.

### Ethical Considerations

Ethics approval was not required for this study because it did not involve human participants, patient data, or animal subjects. The study evaluated outputs from publicly accessible AI tools and internet search engines and therefore did not constitute human subjects research.

### Models

ChatGPT responses were generated using the GPT-4 model via the paid ChatGPT Plus subscription (OpenAI [13]). Google searches were conducted using the Chrome browser.

### Prompt Engineering

Six clinical scenarios representing common presentations of gynecologic cancers were developed by a panel of gynecologic oncology providers. For each cancer type (ovarian, endometrial, and cervical), 7 patient-centered questions were created based on commonly asked topics in clinical practice and information available from professional society websites [14], yielding a total of 21 unique questions (Textbox 1). Questions were entered verbatim into both platforms, with each question generating 1 response from GPT-4 and 1 response from Google. A new ChatGPT session was initiated prior to each question to prevent learning across questions or contextual carryover.

Accuracy was assessed using a 6-point Likert scale: 1=“completely incorrect,” 2=“more incorrect than correct,” 3=“approximately equal incorrect and correct,” 4=“more correct than incorrect,” 5=“nearly all correct,” and 6=“correct.” Completeness was assessed using a 3-point Likert scale: 1=“incomplete,” addressing some aspects of the question but with significant parts missing or incomplete; 2=“adequate,” addressing all aspects of the question and providing the minimum information required to be complete; and 3=“comprehensive,” addressing all aspects of the question and providing additional information or context beyond what was expected. Reference quality was assessed using a 3-point Likert scale: 1=“bottom tier,” including any .com sources such as WebMD; 2=“middle tier,” including any .gov or .org sources such as the American Cancer Society and National Institutes of Health; and 3=“top tier,” including peer-reviewed sources or randomized controlled trials such as the National Comprehensive Cancer Network and UpToDate.

**Clinical questions about diagnosis, treatment options, and survival outcomes for common gynecologic malignancies.**

1. What causes endometrial cancer?
2. How long will I live with stage IB endometrial cancer?
3. What is the recommended treatment for stage IB endometrial cancer?
4. How effective is first-line treatment for stage IB endometrial cancer?
5. How long will I live with stage IIIC endometrial cancer?
6. What is the recommended treatment for stage IIIC endometrial cancer?
7. How effective is first-line treatment for stage IIIC endometrial cancer?
8. What causes cervical cancer?
9. How long will I live with stage IA2 cervical cancer?
10. What is the recommended treatment for stage IA2 cervical cancer?
11. How effective is first-line treatment for stage IA2 cervical cancer?
12. How long will I live with stage IIIC1 cervical cancer?
13. What is the recommended treatment for stage IIIC1 cervical cancer?
14. How effective is first-line treatment for stage IIIC1 cervical cancer?
15. What causes ovarian cancer?
16. How long will I live with stage IC1 ovarian cancer?
17. What is the recommended treatment for stage IC1 ovarian cancer?
18. How effective is first-line treatment for stage IC1 ovarian cancer?
19. How long will I live with stage IVB ovarian cancer?
20. What is the recommended treatment for stage IVB ovarian cancer?
21. How effective is first-line treatment for stage IVB ovarian cancer?

### Query Strategy

Google searches were conducted in private browsing mode with the browser cache cleared. For each query, the top-ranked nonsponsored web page was recorded to allow direct comparison with GPT-4’s single-response output (Multimedia Appendix 1). This approach reflects typical user behavior and aligns with prior studies demonstrating reliance on the first search results [15].

### Performance Evaluation

Responses from both platforms were evaluated for accuracy, completeness, and reference quality by 4 gynecologic oncology physicians from 2 institutions (1 academic tertiary referral center and 1 county-based safety-net hospital). Reviewers represented a range of clinical experience, with 2 physicians having <5 years of clinical experience and 2 having >5 years of clinical experience. Reviewers scored responses independently and were blinded to each other’s evaluations. Reviewers were also invited to provide optional free-text comments within the standardized evaluation form. These qualitative comments were summarized descriptively. Comments were reviewed in their entirety and grouped into thematic categories reflecting perceptions of accuracy, completeness, reference quality, clarity, and patient comprehensibility. A total of 76 optional comments were submitted across all reviewers. Representative comments were selected to illustrate common themes across platforms and are presented in Textbox 2. This descriptive approach aligns with conventional qualitative content analysis methods used to contextualize the quantitative findings [16]. Accuracy was defined as concordance with National Comprehensive Cancer Network guidelines and current literature and scored using a 6-point Likert scale. Completeness was scored using a 3-point Likert scale based on the inclusion of major concepts relevant to each question. Reference quality was categorized as low, medium, or high and scored using a 3-point Likert scale.

**Representative reviewer comments on completeness, accuracy, and reference quality for Google and GPT-4.**

**Google**

- Technically all the information was correct, but it did not answer the specific question. They would likely need to answer this question with another source.
- The question is not answered, only treatments are mentioned. It also makes it sound like the treatments may not be that effective.
- The information looks very accurate, but there is a lot of set up with prognosis and you must scroll down to get your answer.
- Having to weed through the information to get the answer, the information is not as clear and is a little confusing.
- Some information is outdated.
- Very general information which makes it easy for a person with average literacy to understand.
- Answers to survival questions are given but there is no information about the cancer stage.
- There is no mention of treatment effectiveness or immunotherapy.

**GPT-4**

- Accurate information is given that is straight to the point and concise.
- Complete and thorough answers were given that were concise with high quality resources.
- ChatGPT does a nice job of putting information into context for the patient.
- ChatGPT is very comprehensive, although it does not address immunotherapy as an option.
- There is specific data of effectiveness given with very thorough data on treatment options.
- There is very good information provided on surgery and chemotherapy (including details about bevacizumab and PARPi) and also discusses palliative care.
- Information is concise and complete. It talked about what the stage meant.
- My only concern is the patient’s reading level and ability to understand the language in CGPT versus the broad, simple language used by Google.

**Overall**

- I thought I was going to come in biased against ChatGPT. As I looked through the answers, I think ChatGPT did much better answering questions than a random Google search with surprisingly high quality, nuanced answers.
- Google searches with answers from Mayo, COH, ACS were good for basic questions, like what is this cancer, what is the stage; although it gave you all the information on staging and you must scroll to your particular area of interest.
- ChatGPT did a better job at individualizing the answer. It also did better at prognosis, effectiveness questions. It seemed to do it with nuance and “compassion.” But it was also direct, and I think that can be helpful for physicians when it is difficult to give an accurate prognosis.

### Sample Size

A total of 21 standardized patient-centered questions were evaluated. Each question generated 1 response from GPT-4 and 1 response from Google, resulting in 21 responses per platform. All responses were independently evaluated by 4 gynecologic oncology physicians, yielding 84 physician evaluations per platform. The number of questions was selected to capture key informational domains, including etiology, prognosis, treatment, and treatment efficacy, while maintaining feasibility for blinded expert review. This approach is consistent with prior studies evaluating the quality of AI-generated and online health information [8,10,11,17-19].

### Data Analysis

Statistical analyses were conducted using JMP Pro (version 16.0.0; SAS Institute Inc). Descriptive statistics were calculated for all outcomes, and paired comparisons between platforms were performed using Wilcoxon signed-rank tests. Interrater reliability was assessed using intraclass correlation coefficients (ICCs) derived from a 2-way random-effects model with single measures and consistency (ICC[2,1]). Reliability thresholds were interpreted using established criteria [20].

## Results

Accuracy was graded by 4 physicians on a Likert scale of 1 to 6, with 1=“completely incorrect” and 6=“completely correct.” The median score was 5.00 (IQR 4.00-6.00) for Google compared to 6.00 (IQR 5.00-6.00) for GPT-4 (*P*=.04). For Google, the distribution by accuracy score from 1 to 6 was 6% (5/84), 2.4% (2/84), 8.3% (7/84), 16.7% (14/84), 8.3% (7/84), and 58.3% (49/84), respectively. For GPT-4, the distribution from 1 to 6 was 0% (0/84), 0% (0/84), 2.4% (2/84), 10.7% (9/84), 17.8% (15/84), and 69% (58/84), respectively. A score of 5 or 6 (the most accurate) accounted for 66.6% (56/84) of responses for Google compared to 86.9% (73/84) for GPT-4 (Table 1).

Completeness was then graded by 4 physicians on a Likert scale of 1 to 3, with 1=“incomplete,” addressing only some aspects of the questions, and 3=“comprehensive,” addressing all aspects of the questions. The median score was 2.00 (IQR 1.00-3.00) for Google compared to 3.00 (IQR 3.00-3.00) for GPT-4 (*P*=.009). For Google, the distribution by completeness score from 1 to 3 was 36.9% (31/84), 28.6% (24/84), and 34.5% (29/84), respectively. For GPT-4, the distribution from 1 to 3 was 1.2% (1/84), 17.8% (15/84), and 81% (68/84), respectively. A score of 2 or 3 (mostly complete) accounted for 63.1% (53/84) of responses for Google compared to 98.8% (83/84) for GPT-4 (Table 2).

Finally, reference quality was graded by 4 physicians on a 3-point Likert scale, with 1=“bottom tier (.com)” and 3=“top tier (peer reviewed).” The median score was 2.00 (IQR 2.00-2.00) for Google compared to 3.00 (IQR 3.00-3.00) for GPT-4 (*P*=.009). For Google, the distribution by reference quality score from 1 to 3 was 10.7% (9/84), 77.4% (65/84), and 11.9% (10/84), respectively. For GPT-4, the distribution from 1 to 3 was 0% (0/84), 22.6% (19/84), and 77.4% (65/84), respectively. A score of 2 or 3 (highest quality references) accounted for 89.3% (75/84) of responses for Google compared to 100% (84/84) for GPT-4 (Table 3).

Reviewers then provided comments on their perceptions of the accuracy, completeness, and reference quality of GPT-4 compared to Google. A total of 76 optional comments were submitted across all reviewers. Representative comments illustrating the major themes identified across reviewers are presented in Textbox 2. Overall, reviewers noted that Google source responses had clear and precise language but often did not completely answer the question and did not provide enough information on prognosis or treatment options. For GPT-4, reviewers generally commented that answers were very complete and comprehensive, especially regarding disease stage and prognosis, but might be more challenging for patients to understand.

To assess the consistency of reviewer scoring, we calculated interrater reliability using ICCs based on a 2-way random-effects model evaluating consistency. Completeness scores for Google demonstrated moderate consistency (ICC 0.595, 95% CI 0.389-0.782), while all other domains showed poor consistency (ICC<0.50), except for GPT-4 reference quality, which demonstrated excellent interrater reliability (ICC 0.935, 95% CI 0.879-0.970). Full results are shown in Table 4.

**Table 1:** Comparison of accuracy scores for clinical scenarios between GPT-4 and Google.

Measure	Google (n=84)	GPT-4 (n=84)
Accuracy score 1, n (%)	5 (6)	0 (0)
Accuracy score 2, n (%)	2 (2.4)	0 (0)
Accuracy score 3, n (%)	7 (8.3)	2 (2.4)
Accuracy score 4, n (%)	14 (16.7)	9 (10.7)
Accuracy score 5, n (%)	7 (8.3)	15 (17.8)
Accuracy score 6, n (%)	49 (58.3)	58 (69)
Mean (SD)^a^	4.94 (1.51)	5.54 (0.78)
Median (IQR)^a^	6.00 (4.00-6.00)	6.00 (5.00-6.00)

^a^*P*=.04.

**Table 2:** Comparison of completeness scores for clinical scenarios between GPT-4 and Google.

Measure	Google (n=84)	GPT-4 (n=84)
Completeness score 1, n (%)	31 (36.9)	1 (1.2)
Completeness score 2, n (%)	24 (28.6)	15 (17.8)
Completeness score 3, n (%)	29 (34.5)	68 (81)
Mean (SD)^a^	1.98 (0.85)	2.80 (0.43)
Median (IQR)^a^	2.00 (1.00-3.00)	3.00 (3.00-3.00)

^a^*P*=.009.

**Table 3:** Comparison of reference quality scores for clinical scenarios between GPT-4 and Google.

Measure	Google (n=84)	GPT-4 (n=84)
Reference quality score 1, n (%)	9 (10.7)	0 (0)
Reference quality score 2, n (%)	65 (77.4)	19 (22.6)
Reference quality score 3, n (%)	10 (11.9)	65 (77.4)
Mean (SD)^a^	2.01 (0.48)	2.77 (0.42)
Median (IQR)^a^	2.00 (2.00-2.00)	3.00 (3.00-3.00)

^a^*P*=.009.

**Table 4:** Interrater reliability by source and scoring domain.

Source and scoring domains		Interclass correlation^a^ (95% CI)	Interpretation^b^
**Google**			
	Accuracy	0.075 (−0.087 to 0.327)	Poor
	Completeness	0.595 (0.389 to 0.782)	Moderate
	Reference quality	0.016 (−0.128 to 0.255)	Poor
**GPT-4**			
	Accuracy	0.111 (−0.061 to 0369)	Poor
	Completeness	0 (−0.138 to 0.234)	Poor
	Reference quality	0.935 (0.879 to 0.970)	Excellent

^a^Intraclass correlation coefficients were calculated using a 2-way random-effects model with single measures (ICC[2,1]).

^b^Poor<0.5, moderate=0.5-0.75, good=0.75-0.90, and excellent>0.90.

## Discussion

### Principal Results

This study found that GPT-4 performed better than Google sources when providing high-quality responses to clinical scenarios involving a new diagnosis of gynecologic cancer. Physician reviewers rated GPT-4 significantly higher than Google in terms of accuracy, completeness, and reference quality. Reviewers were surprised by the quality of the GPT-4 answers and references and saw the potential for GPT-4 to better address patient-specific questions regarding diagnosis, prognosis, and treatment options with both precise and comprehensive information. The most common shortcoming identified in the Google-sourced content was the provision of general information that was not specific to the patient’s cancer diagnosis, stage, or treatment options and therefore was difficult to individually interpret.

### Comparison With Prior Work

Although a growing number of studies have evaluated GPT-4 for cancer-related patient education, relatively few have focused specifically on gynecologic oncology. Initial research on AI in medicine focused on its diagnostic role and potential for clinical decision-making. For example, in otorhinolaryngology, GPT-4 was compared to UpToDate in terms of usefulness and reliability in diagnosing certain common clinical scenarios. UpToDate was found to be more accurate than GPT-4 (*P*=.009), and the authors concluded that improvements were needed in the usefulness and reliability of GPT-4’s evidence-based knowledge [21]. Similarly, in various clinical cases of thyroid cancer, GPT-4 was found to be reasonably accurate (approximately 76.7%) when providing information about thyroid cancer, but recommendations about evaluation, treatment, and follow-up were deemed general and inadequate [22].

More recently, studies in gynecologic oncology have demonstrated that GPT-4 generally provides guideline-concordant recommendations for gynecologic cancer diagnosis and management, while emphasizing the continued importance of physician oversight for complex clinical decision-making [10,11,23]. Chou et al [17] further reported that GPT-4 generated patient-directed responses regarding ovarian cancer that were comparable to those provided by gynecologic oncology clinicians, supporting its potential role as a patient education tool.

As AI is increasingly explored as a tool for patient-facing communication, several prior studies have evaluated the use of GPT-4 to generate patient information. One study used various LLM platforms to generate patient education materials for both common and rare dermatologic conditions and found that these platforms were able to create accessible, understandable content at an appropriate reading level [18]. Another study focused on the ability of GPT-4 to provide patients with information about screening mammography but documented concerns about the understandability and actionability of the answers, specifically from a health literacy standpoint [19]. Although AI seems to offer an opportunity to enhance patients’ access to health information, there continues to be concern about the possibility of misinformation and the quality of references.

Our findings build upon this growing body of literature. Compared to top-ranked Google search results, GPT-4 consistently outperformed in terms of accuracy, completeness, and reference quality. These findings are consistent with prior studies demonstrating that GPT-4 generally provides accurate, comprehensive, and guideline-concordant information across a variety of cancer-related clinical scenarios [10,11,17,22]. Consistent with the findings of Chou et al [17], we found that GPT-4 generated comprehensive, patient-directed responses that were highly rated by gynecologic oncology physicians. Similarly, our findings align with those of Piazza et al [10] and Finch et al [11], who reported strong agreement between ChatGPT-generated recommendations and established ovarian cancer guidelines. However, although previous studies primarily compared ChatGPT with clinical guidelines or clinician responses, our study directly compared GPT-4 with a conventional Google search strategy, reflecting a common approach used by patients when seeking information after a new cancer diagnosis. Reviewers noted that GPT-4 responses were generally more comprehensive and better aligned with the clinical context, often including relevant treatment information and prognostic details that were lacking in standard search results. Although previous concerns have been raised about the citation quality of previous GPT-4 models, our findings suggest that, in this study, the reference quality was significantly higher than that of Google search results [3,4,8,19,22]. Overall, these results support the potential of GPT-4 to generate personalized, high-quality responses that may enhance patient understanding, support shared decision-making, and improve access to reliable health information.

### Strengths and Limitations

Strengths of this study include the novel design used to investigate the role of AI in generating informational content related to gynecologic malignancies. By querying GPT-4 and Google with multiple patient scenarios, results were more generalizable to patients with different types and stages of gynecologic cancers. The use of gynecologic oncology physicians, who were blinded to each other’s assessments, enabled the clinical evaluation of response quality by experts who routinely guide patients through diagnosis and treatment. Evaluation of accuracy, completeness, and reference quality offered a comprehensive assessment of the content delivered by each platform. Importantly, we also assessed interrater reliability, which is often omitted in similar studies. Although agreement varied by domain, the inclusion of this analysis strengthens the rigor of our evaluation.

Some limitations of this study include a relatively small number of reviewers, a subjective grading scale, and no direct assessment of the readability of the sources used. Although reviewers were blinded to each other’s ratings, they were aware of the source of each response (GPT-4 or Google), which may have introduced bias. Additionally, all 4 reviewers were from only 2 institutions, which raises the possibility that assessments may have been influenced by local practice patterns or institutional standards, potentially limiting generalizability. Interrater reliability was variable across domains, which likely reflects the inherently subjective nature of evaluating informational content, particularly when responses were largely accurate and complete, resulting in clustering of scores at the higher end of the grading scales. In this context, small differences in individual scoring behavior may disproportionately affect interrater reliability metrics despite overall agreement in comparative performance between platforms. Another important consideration is that the phrasing of questions may influence the quality and clarity of responses generated by LLMs. In this study, the scenarios were designed to reflect the types of questions a well-informed patient might ask after receiving a new cancer diagnosis. In real-world settings, however, patients may not know what questions to ask, may lack the background to phrase them effectively, or may not have the clinical knowledge needed to interpret detailed responses. GPT-4 does not tailor responses based on a user’s health literacy level, and this study did not formally assess the readability or accessibility of the responses. This represents an important limitation, as prior research has shown that the effectiveness of patient education materials depends not only on content accuracy but also on whether they are comprehensible and actionable. Additionally, by evaluating only the top-ranked Google result for each query, the study may not fully capture the breadth of information users might access through additional searches.

Since completing this study, Google has expanded the integration of LLM-generated summaries within its search platform. This underscores the rapidly evolving landscape of online health information and highlights the need for continued evaluation of AI-assisted search tools. Although such integrations may narrow performance gaps between traditional search engines and stand-alone LLMs, our findings remain relevant by illustrating the potential advantages of conversational, context-aware responses over static web page retrieval. Future studies should directly compare AI-generated summaries embedded within search engines with stand-alone LLMs, with a focus on reference transparency, patient personalization, and health literacy.

### Conclusions

The results of this study suggest that GPT-4 may be a useful tool for delivering accurate, comprehensive, and well-referenced informational content in gynecologic oncology when compared with top-ranked Google search results within the context of standardized clinical scenarios. However, these findings should be interpreted cautiously given several important methodological limitations, including incomplete blinding of reviewers to response source, variable interrater reliability across evaluation domains, and the restriction of the Google comparator to a single top-ranked result per query. Accordingly, this study should be viewed as a preliminary, hypothesis-generating evaluation rather than a definitive assessment of comparative performance. Although LLMs such as GPT-4 show promise in generating context-aware responses tailored to specific clinical scenarios, further research is needed to clarify their appropriate role in supporting patient education and health care professional–patient communication in gynecologic oncology.

## Appendix

Multimedia Appendix 1 Gynecologic oncology case questions, source materials, and physician grading rubrics used to evaluate Google and GPT-4 responses.

## Abbreviations

CHART: Chatbot Health Advice Reporting Tool
ICC: intraclass correlation coefficient
LLM: large language model
