# Supplementary material for: Assessing the Accuracy, Completeness, and Reference Quality of GPT-4 and Google for Gynecologic Cancer Information: Comparative Quantitative Study
Source: JMIR Cancer. 2026 Sep 29;12:e76471. doi: 10.2196/76471 (PMC13662441; doi:10.2196/76471)
Supplement: Supplementary file 1 [file cancer_v12i1e76471_app1.docx]

**Supplemental Data**

**S1. Case questions and associated patient education materials.**

**Endometrial cancer:**

Q1: What causes endometrial cancer?

Google: <https://www.mayoclinic.org/diseases-conditions/endometrial-cancer/symptoms-causes/syc-20352461>

ChatGPT: <https://chatgpt.com/share/66e47461-79ec-8002-a68c-9940fe823114>

**Case 1: Stage IB endometrial cancer**

Q2: How long will I live with stage IB endometrial cancer?

Google: <https://www.saintlukeskc.org/health-library/endometrial-cancer-your-chances-recovery-prognosis>

ChatGPT: <https://chatgpt.com/share/66e474cd-bf74-8002-ad71-c00220aa48d3>

Q3: What is the recommended treatment for stage IB endometrial cancer?

Google: <https://www.cancer.org/cancer/types/endometrial-cancer/treating/by-stage.html>

ChatGPT: <https://chatgpt.com/share/66e47559-500c-8002-bbd9-4abbba0d5263>

Q4: How effective is first line treatment for stage IB endometrial cancer?

Google: <https://www.cancer.org/cancer/types/endometrial-cancer/treating/by-stage.html>

ChatGPT: <https://chatgpt.com/share/66e475d7-c5dc-8002-8238-3f3a80e929fa>

**Case 2: Stage IIIC endometrial cancer**

Q5: How long will I live with stage IIIC endometrial cancer?

Google: <https://www.saintlukeskc.org/health-library/endometrial-cancer-your-chances-recovery-prognosis>

ChatGPT: <https://chatgpt.com/share/66e47693-85c0-8002-822b-bd033e80ceff>

Q6: What is the recommended treatment for stage IIIC endometrial cancer?

Google: <https://www.cancer.org/cancer/types/endometrial-cancer/treating/by-stage.html>

ChatGPT: <https://chatgpt.com/share/66e476f1-c4e8-8002-839b-30b44a9132b3>

Q7: How effective is first line treatment for stage IIIC endometrial cancer?

Google: <https://www.cancer.org/cancer/types/endometrial-cancer/treating/by-stage.html>

ChatGPT: <https://chatgpt.com/share/66e477a3-6d5c-8002-aff3-f4da04839050>

**Cervical cancer:**

Q8: What causes cervical cancer?

Google: <https://www.mayoclinic.org/diseases-conditions/cervical-cancer/symptoms-causes/syc-20352501>

ChatGPT: <https://chatgpt.com/share/66e4784c-088c-8002-835b-b5a37e45cc9c>

**Case 3: Stage IA2 cervical cancer**

Q9: How long will I live with stage IA2 cervical cancer?

Google: <https://www.cancer.gov/types/cervical/survival>

ChatGPT: <https://chatgpt.com/share/66e478d7-0f74-8002-b65a-f842d03b3bdd>

Q10: What is the recommended treatment for stage IA2 cervical cancer?

Google: <https://www.cancer.org/cancer/types/cervical-cancer/treating/by-stage.html>

ChatGPT: <https://chatgpt.com/share/66e4791c-2864-8002-b3c4-3255a9304a2e>

Q11: How effective is first line treatment for stage IA2 cervical cancer?

Google: <https://www.cancer.org/cancer/types/cervical-cancer/treating/by-stage.html>

ChatGPT: <https://chatgpt.com/share/66e4797e-d684-8002-baae-74aa45ebf1ce>

**Case 4: Stage IIIC1 cervical cancer**

Q12: How long will I live with stage IIIC1 cervical cancer?

Google: <https://www.cancer.gov/types/cervical/survival>

ChatGPT: <https://chatgpt.com/share/66e47a29-1254-8002-b9f9-6c4ff49f3958>

Q13: What is my recommended treatment for stage IIIC1 cervical cancer?

Google: <https://www.cancer.org/cancer/types/cervical-cancer/treating/by-stage.html>

ChatGPT: <https://chatgpt.com/share/66e47b0a-3548-8002-9798-40b687a8f27a>

Q14: How effective is first line treatment for stage IIIC1 cervical cancer?

Google: https://www.cancer.gov/types/cervical/treatment/by-stage

ChatGPT: <https://chatgpt.com/share/66e47ba8-7404-8002-9f7f-2e2e5cc20a47>

**Ovarian cancer:**

Q15: What causes ovarian cancer?

Google: <https://www.mayoclinic.org/diseases-conditions/ovarian-cancer/symptoms-causes/syc-20375941>

ChatGPT: <https://chatgpt.com/share/66e47ce7-9ae0-8002-afc0-710acaf333f4>

**Case 5: Stage IC1 ovarian cancer**

Q16: How long will I live with stage IC1 ovarian cancer?

Google: <https://www.cancercenter.com/cancer-types/ovarian-cancer/stages>

ChatGPT: <https://chatgpt.com/share/66e4a053-0174-8002-a48d-66805093da3c>

Q17: What is the recommended treatment for stage IC1 ovarian cancer?

Google: <https://www.cancer.org/cancer/types/ovarian-cancer/treating/by-stage.html>

ChatGPT: <https://chatgpt.com/share/66e4a08f-75e4-8002-837e-83cc2a29f3be>

Q18: How effective is first line treatment for stage IC1 ovarian cancer?

Google: https://www.cancer.org/cancer/types/ovarian-cancer/treating/by-stage.html

ChatGPT: <https://chatgpt.com/share/66e4a0d5-5b60-8002-9b44-3757f4031ede>

**Case 6: Stage IVB ovarian cancer**

Q19: How long will I live with stage IVB ovarian cancer?

Google: <https://ocrahope.org/get-the-facts/staging/>

ChatGPT: <https://chatgpt.com/share/66e4a155-4f7c-8002-9e91-387e347c1337>

Q20: What is the recommended treatment for stage IVB ovarian cancer?

Google: <https://www.cancer.org/cancer/types/ovarian-cancer/treating/by-stage.html>

ChatGPT: <https://chatgpt.com/share/66e4a1b2-8100-8002-b1e5-21a026be18c9>

Q21: How effective is first line treatment for stage IVB ovarian cancer?

Google: <https://www.cancer.org/cancer/types/ovarian-cancer/treating/by-stage.html>

ChatGPT: <https://chatgpt.com/share/66e4a29e-6940-8002-9e03-26083099bcf2>

**S2. Grading rubrics by case question.**

**Endometrial cancer:**

Q1: What causes endometrial cancer?

**Google:**

| Accuracy | 1 | 2 | 3 | 4 | 5 | 6 |
| --- | --- | --- | --- | --- | --- | --- |
|  | Completely incorrect | More incorrect than correct | Approximately equal incorrect and correct | More correct than incorrect | Nearly all correct | Correct |

| Completeness | 1 | 2 | 3 |
| --- | --- | --- | --- |
|  | incomplete, addresses some  aspects of the question, but significant parts are missing or incomplete | adequate, addresses all aspects of the question and provides the minimum  amount of information required to be considered complete | comprehensive, addresses all aspects of the question and provides additional information or  context beyond what was expected |

| Reference | 1 | 2 | 3 |
| --- | --- | --- | --- |
|  | Bottom tier: Any .com (webMD, etc) | Middle tier: Any .gov or .org (ACS, NIH, etc) | Top tier: Peer reviewed or RCT (NCCN, uptodate, etc.) |

Comments:

**ChatGPT:**

| Accuracy | 1 | 2 | 3 | 4 | 5 | 6 |
| --- | --- | --- | --- | --- | --- | --- |
|  | Completely incorrect | More incorrect than correct | Approximately equal incorrect and correct | More correct than incorrect | Nearly all correct | Correct |

| Completeness | 1 | 2 | 3 |
| --- | --- | --- | --- |
|  | incomplete, addresses some  aspects of the question, but significant parts are missing or incomplete | adequate, addresses all aspects of the question and provides the minimum  amount of information required to be considered complete | comprehensive, addresses all aspects of the question and provides additional information or  context beyond what was expected |

| Reference | 1 | 2 | 3 |
| --- | --- | --- | --- |
|  | Bottom tier: Any .com (webMD, etc) | Middle tier: Any .gov or .org (ACS, NIH, etc) | Top tier: Peer reviewed or RCT (NCCN, uptodate, etc.) |

Comments:

**Case 1: Stage IB endometrial cancer**

Q2: How long will I live with stage IB endometrial cancer?

**Google:**

| Accuracy | 1 | 2 | 3 | 4 | 5 | 6 |
| --- | --- | --- | --- | --- | --- | --- |
|  | Completely incorrect | More incorrect than correct | Approximately equal incorrect and correct | More correct than incorrect | Nearly all correct | Correct |

| Completeness | 1 | 2 | 3 |
| --- | --- | --- | --- |
|  | incomplete, addresses some  aspects of the question, but significant parts are missing or incomplete | adequate, addresses all aspects of the question and provides the minimum  amount of information required to be considered complete | comprehensive, addresses all aspects of the question and provides additional information or  context beyond what was expected |

| Reference | 1 | 2 | 3 |
| --- | --- | --- | --- |
|  | Bottom tier: Any .com (webMD, etc) | Middle tier: Any .gov or .org (ACS, NIH, etc) | Top tier: Peer reviewed or RCT (NCCN, uptodate, etc.) |

Comments:

**ChatGPT:**

| Accuracy | 1 | 2 | 3 | 4 | 5 | 6 |
| --- | --- | --- | --- | --- | --- | --- |
|  | Completely incorrect | More incorrect than correct | Approximately equal incorrect and correct | More correct than incorrect | Nearly all correct | Correct |

| Completeness | 1 | 2 | 3 |
| --- | --- | --- | --- |
|  | incomplete, addresses some  aspects of the question, but significant parts are missing or incomplete | adequate, addresses all aspects of the question and provides the minimum  amount of information required to be considered complete | comprehensive, addresses all aspects of the question and provides additional information or  context beyond what was expected |

| Reference | 1 | 2 | 3 |
| --- | --- | --- | --- |
|  | Bottom tier: Any .com (webMD, etc) | Middle tier: Any .gov or .org (ACS, NIH, etc) | Top tier: Peer reviewed or RCT (NCCN, uptodate, etc.) |

Comments:

Q3: What is the recommended treatment for stage IB endometrial cancer?

**Google:**

| Accuracy | 1 | 2 | 3 | 4 | 5 | 6 |
| --- | --- | --- | --- | --- | --- | --- |
|  | Completely incorrect | More incorrect than correct | Approximately equal incorrect and correct | More correct than incorrect | Nearly all correct | Correct |

| Completeness | 1 | 2 | 3 |
| --- | --- | --- | --- |
|  | incomplete, addresses some  aspects of the question, but significant parts are missing or incomplete | adequate, addresses all aspects of the question and provides the minimum  amount of information required to be considered complete | comprehensive, addresses all aspects of the question and provides additional information or  context beyond what was expected |

| Reference | 1 | 2 | 3 |
| --- | --- | --- | --- |
|  | Bottom tier: Any .com (webMD, etc) | Middle tier: Any .gov or .org (ACS, NIH, etc) | Top tier: Peer reviewed or RCT (NCCN, uptodate, etc.) |

Comments:

**ChatGPT:**

| Accuracy | 1 | 2 | 3 | 4 | 5 | 6 |
| --- | --- | --- | --- | --- | --- | --- |
|  | Completely incorrect | More incorrect than correct | Approximately equal incorrect and correct | More correct than incorrect | Nearly all correct | Correct |

| Completeness | 1 | 2 | 3 |
| --- | --- | --- | --- |
|  | incomplete, addresses some  aspects of the question, but significant parts are missing or incomplete | adequate, addresses all aspects of the question and provides the minimum  amount of information required to be considered complete | comprehensive, addresses all aspects of the question and provides additional information or  context beyond what was expected |

| Reference | 1 | 2 | 3 |
| --- | --- | --- | --- |
|  | Bottom tier: Any .com (webMD, etc) | Middle tier: Any .gov or .org (ACS, NIH, etc) | Top tier: Peer reviewed or RCT (NCCN, uptodate, etc.) |

Comments:

Q4: How effective is first line treatment for stage IB endometrial cancer?

**Google:**

| Accuracy | 1 | 2 | 3 | 4 | 5 | 6 |
| --- | --- | --- | --- | --- | --- | --- |
|  | Completely incorrect | More incorrect than correct | Approximately equal incorrect and correct | More correct than incorrect | Nearly all correct | Correct |

| Completeness | 1 | 2 | 3 |
| --- | --- | --- | --- |
|  | incomplete, addresses some  aspects of the question, but significant parts are missing or incomplete | adequate, addresses all aspects of the question and provides the minimum  amount of information required to be considered complete | comprehensive, addresses all aspects of the question and provides additional information or  context beyond what was expected |

| Reference | 1 | 2 | 3 |
| --- | --- | --- | --- |
|  | Bottom tier: Any .com (webMD, etc) | Middle tier: Any .gov or .org (ACS, NIH, etc) | Top tier: Peer reviewed or RCT (NCCN, uptodate, etc.) |

Comments:

**ChatGPT:**

| Accuracy | 1 | 2 | 3 | 4 | 5 | 6 |
| --- | --- | --- | --- | --- | --- | --- |
|  | Completely incorrect | More incorrect than correct | Approximately equal incorrect and correct | More correct than incorrect | Nearly all correct | Correct |

| Completeness | 1 | 2 | 3 |
| --- | --- | --- | --- |
|  | incomplete, addresses some  aspects of the question, but significant parts are missing or incomplete | adequate, addresses all aspects of the question and provides the minimum  amount of information required to be considered complete | comprehensive, addresses all aspects of the question and provides additional information or  context beyond what was expected |

| Reference | 1 | 2 | 3 |
| --- | --- | --- | --- |
|  | Bottom tier: Any .com (webMD, etc) | Middle tier: Any .gov or .org (ACS, NIH, etc) | Top tier: Peer reviewed or RCT (NCCN, uptodate, etc.) |

Comments:

**Case 2: Stage IIIC endometrial cancer**

Q5: How long will I live with stage IIIC endometrial cancer?

**Google:**

| Accuracy | 1 | 2 | 3 | 4 | 5 | 6 |
| --- | --- | --- | --- | --- | --- | --- |
|  | Completely incorrect | More incorrect than correct | Approximately equal incorrect and correct | More correct than incorrect | Nearly all correct | Correct |

| Completeness | 1 | 2 | 3 |
| --- | --- | --- | --- |
|  | incomplete, addresses some  aspects of the question, but significant parts are missing or incomplete | adequate, addresses all aspects of the question and provides the minimum  amount of information required to be considered complete | comprehensive, addresses all aspects of the question and provides additional information or  context beyond what was expected |

| Reference | 1 | 2 | 3 |
| --- | --- | --- | --- |
|  | Bottom tier: Any .com (webMD, etc) | Middle tier: Any .gov or .org (ACS, NIH, etc) | Top tier: Peer reviewed or RCT (NCCN, uptodate, etc.) |

Comments:

**ChatGPT:**

| Accuracy | 1 | 2 | 3 | 4 | 5 | 6 |
| --- | --- | --- | --- | --- | --- | --- |
|  | Completely incorrect | More incorrect than correct | Approximately equal incorrect and correct | More correct than incorrect | Nearly all correct | Correct |

| Completeness | 1 | 2 | 3 |
| --- | --- | --- | --- |
|  | incomplete, addresses some  aspects of the question, but significant parts are missing or incomplete | adequate, addresses all aspects of the question and provides the minimum  amount of information required to be considered complete | comprehensive, addresses all aspects of the question and provides additional information or  context beyond what was expected |

| Reference | 1 | 2 | 3 |
| --- | --- | --- | --- |
|  | Bottom tier: Any .com (webMD, etc) | Middle tier: Any .gov or .org (ACS, NIH, etc) | Top tier: Peer reviewed or RCT (NCCN, uptodate, etc.) |

Comments:

Q6: What is the recommended treatment for stage IIIC endometrial cancer?

**Google:**

| Accuracy | 1 | 2 | 3 | 4 | 5 | 6 |
| --- | --- | --- | --- | --- | --- | --- |
|  | Completely incorrect | More incorrect than correct | Approximately equal incorrect and correct | More correct than incorrect | Nearly all correct | Correct |

| Completeness | 1 | 2 | 3 |
| --- | --- | --- | --- |
|  | incomplete, addresses some  aspects of the question, but significant parts are missing or incomplete | adequate, addresses all aspects of the question and provides the minimum  amount of information required to be considered complete | comprehensive, addresses all aspects of the question and provides additional information or  context beyond what was expected |

| Reference | 1 | 2 | 3 |
| --- | --- | --- | --- |
|  | Bottom tier: Any .com (webMD, etc) | Middle tier: Any .gov or .org (ACS, NIH, etc) | Top tier: Peer reviewed or RCT (NCCN, uptodate, etc.) |

Comments:

**ChatGPT:**

| Accuracy | 1 | 2 | 3 | 4 | 5 | 6 |
| --- | --- | --- | --- | --- | --- | --- |
|  | Completely incorrect | More incorrect than correct | Approximately equal incorrect and correct | More correct than incorrect | Nearly all correct | Correct |

| Completeness | 1 | 2 | 3 |
| --- | --- | --- | --- |
|  | incomplete, addresses some  aspects of the question, but significant parts are missing or incomplete | adequate, addresses all aspects of the question and provides the minimum  amount of information required to be considered complete | comprehensive, addresses all aspects of the question and provides additional information or  context beyond what was expected |

| Reference | 1 | 2 | 3 |
| --- | --- | --- | --- |
|  | Bottom tier: Any .com (webMD, etc) | Middle tier: Any .gov or .org (ACS, NIH, etc) | Top tier: Peer reviewed or RCT (NCCN, uptodate, etc.) |

Comments:

Q7: How effective is first line treatment for stage IIIC endometrial cancer?

**Google:**

| Accuracy | 1 | 2 | 3 | 4 | 5 | 6 |
| --- | --- | --- | --- | --- | --- | --- |
|  | Completely incorrect | More incorrect than correct | Approximately equal incorrect and correct | More correct than incorrect | Nearly all correct | Correct |

| Completeness | 1 | 2 | 3 |
| --- | --- | --- | --- |
|  | incomplete, addresses some  aspects of the question, but significant parts are missing or incomplete | adequate, addresses all aspects of the question and provides the minimum  amount of information required to be considered complete | comprehensive, addresses all aspects of the question and provides additional information or  context beyond what was expected |

| Reference | 1 | 2 | 3 |
| --- | --- | --- | --- |
|  | Bottom tier: Any .com (webMD, etc) | Middle tier: Any .gov or .org (ACS, NIH, etc) | Top tier: Peer reviewed or RCT (NCCN, uptodate, etc.) |

Comments:

**ChatGPT:**

| Accuracy | 1 | 2 | 3 | 4 | 5 | 6 |
| --- | --- | --- | --- | --- | --- | --- |
|  | Completely incorrect | More incorrect than correct | Approximately equal incorrect and correct | More correct than incorrect | Nearly all correct | Correct |

| Completeness | 1 | 2 | 3 |
| --- | --- | --- | --- |
|  | incomplete, addresses some  aspects of the question, but significant parts are missing or incomplete | adequate, addresses all aspects of the question and provides the minimum  amount of information required to be considered complete | comprehensive, addresses all aspects of the question and provides additional information or  context beyond what was expected |

| Reference | 1 | 2 | 3 |
| --- | --- | --- | --- |
|  | Bottom tier: Any .com (webMD, etc) | Middle tier: Any .gov or .org (ACS, NIH, etc) | Top tier: Peer reviewed or RCT (NCCN, uptodate, etc.) |

Comments:

**Cervical cancer:**

Q8: What causes cervical cancer?

**Google:**

| Accuracy | 1 | 2 | 3 | 4 | 5 | 6 |
| --- | --- | --- | --- | --- | --- | --- |
|  | Completely incorrect | More incorrect than correct | Approximately equal incorrect and correct | More correct than incorrect | Nearly all correct | Correct |

| Completeness | 1 | 2 | 3 |
| --- | --- | --- | --- |
|  | incomplete, addresses some  aspects of the question, but significant parts are missing or incomplete | adequate, addresses all aspects of the question and provides the minimum  amount of information required to be considered complete | comprehensive, addresses all aspects of the question and provides additional information or  context beyond what was expected |

| Reference | 1 | 2 | 3 |
| --- | --- | --- | --- |
|  | Bottom tier: Any .com (webMD, etc) | Middle tier: Any .gov or .org (ACS, NIH, etc) | Top tier: Peer reviewed or RCT (NCCN, uptodate, etc.) |

Comments:

**ChatGPT:**

| Accuracy | 1 | 2 | 3 | 4 | 5 | 6 |
| --- | --- | --- | --- | --- | --- | --- |
|  | Completely incorrect | More incorrect than correct | Approximately equal incorrect and correct | More correct than incorrect | Nearly all correct | Correct |

| Completeness | 1 | 2 | 3 |
| --- | --- | --- | --- |
|  | incomplete, addresses some  aspects of the question, but significant parts are missing or incomplete | adequate, addresses all aspects of the question and provides the minimum  amount of information required to be considered complete | comprehensive, addresses all aspects of the question and provides additional information or  context beyond what was expected |

| Reference | 1 | 2 | 3 |
| --- | --- | --- | --- |
|  | Bottom tier: Any .com (webMD, etc) | Middle tier: Any .gov or .org (ACS, NIH, etc) | Top tier: Peer reviewed or RCT (NCCN, uptodate, etc.) |

Comments:

**Case 3: Stage IA2 cervical cancer**

Q9: How long will I live with stage IA2 cervical cancer?

**Google:**

| Accuracy | 1 | 2 | 3 | 4 | 5 | 6 |
| --- | --- | --- | --- | --- | --- | --- |
|  | Completely incorrect | More incorrect than correct | Approximately equal incorrect and correct | More correct than incorrect | Nearly all correct | Correct |

| Completeness | 1 | 2 | 3 |
| --- | --- | --- | --- |
|  | incomplete, addresses some  aspects of the question, but significant parts are missing or incomplete | adequate, addresses all aspects of the question and provides the minimum  amount of information required to be considered complete | comprehensive, addresses all aspects of the question and provides additional information or  context beyond what was expected |

| Reference | 1 | 2 | 3 |
| --- | --- | --- | --- |
|  | Bottom tier: Any .com (webMD, etc) | Middle tier: Any .gov or .org (ACS, NIH, etc) | Top tier: Peer reviewed or RCT (NCCN, uptodate, etc.) |

Comments:

**ChatGPT:**

| Accuracy | 1 | 2 | 3 | 4 | 5 | 6 |
| --- | --- | --- | --- | --- | --- | --- |
|  | Completely incorrect | More incorrect than correct | Approximately equal incorrect and correct | More correct than incorrect | Nearly all correct | Correct |

| Completeness | 1 | 2 | 3 |
| --- | --- | --- | --- |
|  | incomplete, addresses some  aspects of the question, but significant parts are missing or incomplete | adequate, addresses all aspects of the question and provides the minimum  amount of information required to be considered complete | comprehensive, addresses all aspects of the question and provides additional information or  context beyond what was expected |

| Reference | 1 | 2 | 3 |
| --- | --- | --- | --- |
|  | Bottom tier: Any .com (webMD, etc) | Middle tier: Any .gov or .org (ACS, NIH, etc) | Top tier: Peer reviewed or RCT (NCCN, uptodate, etc.) |

Comments:

Q10: What is the recommended treatment for stage IA2 cervical cancer?

**Google:**

| Accuracy | 1 | 2 | 3 | 4 | 5 | 6 |
| --- | --- | --- | --- | --- | --- | --- |
|  | Completely incorrect | More incorrect than correct | Approximately equal incorrect and correct | More correct than incorrect | Nearly all correct | Correct |

| Completeness | 1 | 2 | 3 |
| --- | --- | --- | --- |
|  | incomplete, addresses some  aspects of the question, but significant parts are missing or incomplete | adequate, addresses all aspects of the question and provides the minimum  amount of information required to be considered complete | comprehensive, addresses all aspects of the question and provides additional information or  context beyond what was expected |

| Reference | 1 | 2 | 3 |
| --- | --- | --- | --- |
|  | Bottom tier: Any .com (webMD, etc) | Middle tier: Any .gov or .org (ACS, NIH, etc) | Top tier: Peer reviewed or RCT (NCCN, uptodate, etc.) |

Comments:

**ChatGPT:**

| Accuracy | 1 | 2 | 3 | 4 | 5 | 6 |
| --- | --- | --- | --- | --- | --- | --- |
|  | Completely incorrect | More incorrect than correct | Approximately equal incorrect and correct | More correct than incorrect | Nearly all correct | Correct |

| Completeness | 1 | 2 | 3 |
| --- | --- | --- | --- |
|  | incomplete, addresses some  aspects of the question, but significant parts are missing or incomplete | adequate, addresses all aspects of the question and provides the minimum  amount of information required to be considered complete | comprehensive, addresses all aspects of the question and provides additional information or  context beyond what was expected |

| Reference | 1 | 2 | 3 |
| --- | --- | --- | --- |
|  | Bottom tier: Any .com (webMD, etc) | Middle tier: Any .gov or .org (ACS, NIH, etc) | Top tier: Peer reviewed or RCT (NCCN, uptodate, etc.) |

Comments:

Q11: How effective is first line treatment for stage IA2 cervical cancer?

**Google:**

| Accuracy | 1 | 2 | 3 | 4 | 5 | 6 |
| --- | --- | --- | --- | --- | --- | --- |
|  | Completely incorrect | More incorrect than correct | Approximately equal incorrect and correct | More correct than incorrect | Nearly all correct | Correct |

| Completeness | 1 | 2 | 3 |
| --- | --- | --- | --- |
|  | incomplete, addresses some  aspects of the question, but significant parts are missing or incomplete | adequate, addresses all aspects of the question and provides the minimum  amount of information required to be considered complete | comprehensive, addresses all aspects of the question and provides additional information or  context beyond what was expected |

| Reference | 1 | 2 | 3 |
| --- | --- | --- | --- |
|  | Bottom tier: Any .com (webMD, etc) | Middle tier: Any .gov or .org (ACS, NIH, etc) | Top tier: Peer reviewed or RCT (NCCN, uptodate, etc.) |

Comments:

**ChatGPT:**

| Accuracy | 1 | 2 | 3 | 4 | 5 | 6 |
| --- | --- | --- | --- | --- | --- | --- |
|  | Completely incorrect | More incorrect than correct | Approximately equal incorrect and correct | More correct than incorrect | Nearly all correct | Correct |

| Completeness | 1 | 2 | 3 |
| --- | --- | --- | --- |
|  | incomplete, addresses some  aspects of the question, but significant parts are missing or incomplete | adequate, addresses all aspects of the question and provides the minimum  amount of information required to be considered complete | comprehensive, addresses all aspects of the question and provides additional information or  context beyond what was expected |

| Reference | 1 | 2 | 3 |
| --- | --- | --- | --- |
|  | Bottom tier: Any .com (webMD, etc) | Middle tier: Any .gov or .org (ACS, NIH, etc) | Top tier: Peer reviewed or RCT (NCCN, uptodate, etc.) |

Comments:

**Case 4: Stage IIIC1 cervical cancer**

Q12: How long will I live with stage IIIC1 cervical cancer?

**Google:**

| Accuracy | 1 | 2 | 3 | 4 | 5 | 6 |
| --- | --- | --- | --- | --- | --- | --- |
|  | Completely incorrect | More incorrect than correct | Approximately equal incorrect and correct | More correct than incorrect | Nearly all correct | Correct |

| Completeness | 1 | 2 | 3 |
| --- | --- | --- | --- |
|  | incomplete, addresses some  aspects of the question, but significant parts are missing or incomplete | adequate, addresses all aspects of the question and provides the minimum  amount of information required to be considered complete | comprehensive, addresses all aspects of the question and provides additional information or  context beyond what was expected |

| Reference | 1 | 2 | 3 |
| --- | --- | --- | --- |
|  | Bottom tier: Any .com (webMD, etc) | Middle tier: Any .gov or .org (ACS, NIH, etc) | Top tier: Peer reviewed or RCT (NCCN, uptodate, etc.) |

Comments:

**ChatGPT:**

| Accuracy | 1 | 2 | 3 | 4 | 5 | 6 |
| --- | --- | --- | --- | --- | --- | --- |
|  | Completely incorrect | More incorrect than correct | Approximately equal incorrect and correct | More correct than incorrect | Nearly all correct | Correct |

| Completeness | 1 | 2 | 3 |
| --- | --- | --- | --- |
|  | incomplete, addresses some  aspects of the question, but significant parts are missing or incomplete | adequate, addresses all aspects of the question and provides the minimum  amount of information required to be considered complete | comprehensive, addresses all aspects of the question and provides additional information or  context beyond what was expected |

| Reference | 1 | 2 | 3 |
| --- | --- | --- | --- |
|  | Bottom tier: Any .com (webMD, etc) | Middle tier: Any .gov or .org (ACS, NIH, etc) | Top tier: Peer reviewed or RCT (NCCN, uptodate, etc.) |

Comments:

Q13: What is my recommended treatment for stage IIIC1 cervical cancer?

**Google:**

| Accuracy | 1 | 2 | 3 | 4 | 5 | 6 |
| --- | --- | --- | --- | --- | --- | --- |
|  | Completely incorrect | More incorrect than correct | Approximately equal incorrect and correct | More correct than incorrect | Nearly all correct | Correct |

| Completeness | 1 | 2 | 3 |
| --- | --- | --- | --- |
|  | incomplete, addresses some  aspects of the question, but significant parts are missing or incomplete | adequate, addresses all aspects of the question and provides the minimum  amount of information required to be considered complete | comprehensive, addresses all aspects of the question and provides additional information or  context beyond what was expected |

| Reference | 1 | 2 | 3 |
| --- | --- | --- | --- |
|  | Bottom tier: Any .com (webMD, etc) | Middle tier: Any .gov or .org (ACS, NIH, etc) | Top tier: Peer reviewed or RCT (NCCN, uptodate, etc.) |

Comments:

**ChatGPT:**

| Accuracy | 1 | 2 | 3 | 4 | 5 | 6 |
| --- | --- | --- | --- | --- | --- | --- |
|  | Completely incorrect | More incorrect than correct | Approximately equal incorrect and correct | More correct than incorrect | Nearly all correct | Correct |

| Completeness | 1 | 2 | 3 |
| --- | --- | --- | --- |
|  | incomplete, addresses some  aspects of the question, but significant parts are missing or incomplete | adequate, addresses all aspects of the question and provides the minimum  amount of information required to be considered complete | comprehensive, addresses all aspects of the question and provides additional information or  context beyond what was expected |

| Reference | 1 | 2 | 3 |
| --- | --- | --- | --- |
|  | Bottom tier: Any .com (webMD, etc) | Middle tier: Any .gov or .org (ACS, NIH, etc) | Top tier: Peer reviewed or RCT (NCCN, uptodate, etc.) |

Comments:

Q14: How effective is first line treatment for stage IIIC1 cervical cancer?

**Google:**

| Accuracy | 1 | 2 | 3 | 4 | 5 | 6 |
| --- | --- | --- | --- | --- | --- | --- |
|  | Completely incorrect | More incorrect than correct | Approximately equal incorrect and correct | More correct than incorrect | Nearly all correct | Correct |

| Completeness | 1 | 2 | 3 |
| --- | --- | --- | --- |
|  | incomplete, addresses some  aspects of the question, but significant parts are missing or incomplete | adequate, addresses all aspects of the question and provides the minimum  amount of information required to be considered complete | comprehensive, addresses all aspects of the question and provides additional information or  context beyond what was expected |

| Reference | 1 | 2 | 3 |
| --- | --- | --- | --- |
|  | Bottom tier: Any .com (webMD, etc) | Middle tier: Any .gov or .org (ACS, NIH, etc) | Top tier: Peer reviewed or RCT (NCCN, uptodate, etc.) |

**Comments:**

**ChatGPT:**

| Accuracy | 1 | 2 | 3 | 4 | 5 | 6 |
| --- | --- | --- | --- | --- | --- | --- |
|  | Completely incorrect | More incorrect than correct | Approximately equal incorrect and correct | More correct than incorrect | Nearly all correct | Correct |

| Completeness | 1 | 2 | 3 |
| --- | --- | --- | --- |
|  | incomplete, addresses some  aspects of the question, but significant parts are missing or incomplete | adequate, addresses all aspects of the question and provides the minimum  amount of information required to be considered complete | comprehensive, addresses all aspects of the question and provides additional information or  context beyond what was expected |

| Reference | 1 | 2 | 3 |
| --- | --- | --- | --- |
|  | Bottom tier: Any .com (webMD, etc) | Middle tier: Any .gov or .org (ACS, NIH, etc) | Top tier: Peer reviewed or RCT (NCCN, uptodate, etc.) |

Comments:

**Ovarian cancer:**

Q15: What causes ovarian cancer?

**Google:**

| Accuracy | 1 | 2 | 3 | 4 | 5 | 6 |
| --- | --- | --- | --- | --- | --- | --- |
|  | Completely incorrect | More incorrect than correct | Approximately equal incorrect and correct | More correct than incorrect | Nearly all correct | Correct |

| Completeness | 1 | 2 | 3 |
| --- | --- | --- | --- |
|  | incomplete, addresses some  aspects of the question, but significant parts are missing or incomplete | adequate, addresses all aspects of the question and provides the minimum  amount of information required to be considered complete | comprehensive, addresses all aspects of the question and provides additional information or  context beyond what was expected |

| Reference | 1 | 2 | 3 |
| --- | --- | --- | --- |
|  | Bottom tier: Any .com (webMD, etc) | Middle tier: Any .gov or .org (ACS, NIH, etc) | Top tier: Peer reviewed or RCT (NCCN, uptodate, etc.) |

Comments:

**ChatGPT:**

| Accuracy | 1 | 2 | 3 | 4 | 5 | 6 |
| --- | --- | --- | --- | --- | --- | --- |
|  | Completely incorrect | More incorrect than correct | Approximately equal incorrect and correct | More correct than incorrect | Nearly all correct | Correct |

| Completeness | 1 | 2 | 3 |
| --- | --- | --- | --- |
|  | incomplete, addresses some  aspects of the question, but significant parts are missing or incomplete | adequate, addresses all aspects of the question and provides the minimum  amount of information required to be considered complete | comprehensive, addresses all aspects of the question and provides additional information or  context beyond what was expected |

| Reference | 1 | 2 | 3 |
| --- | --- | --- | --- |
|  | Bottom tier: Any .com (webMD, etc) | Middle tier: Any .gov or .org (ACS, NIH, etc) | Top tier: Peer reviewed or RCT (NCCN, uptodate, etc.) |

Comments:

**Case 5: Stage IC1 ovarian cancer**

Q16: How long will I live with stage IC1 ovarian cancer?

**Google:**

| Accuracy | 1 | 2 | 3 | 4 | 5 | 6 |
| --- | --- | --- | --- | --- | --- | --- |
|  | Completely incorrect | More incorrect than correct | Approximately equal incorrect and correct | More correct than incorrect | Nearly all correct | Correct |

| Completeness | 1 | 2 | 3 |
| --- | --- | --- | --- |
|  | incomplete, addresses some  aspects of the question, but significant parts are missing or incomplete | adequate, addresses all aspects of the question and provides the minimum  amount of information required to be considered complete | comprehensive, addresses all aspects of the question and provides additional information or  context beyond what was expected |

| Reference | 1 | 2 | 3 |
| --- | --- | --- | --- |
|  | Bottom tier: Any .com (webMD, etc) | Middle tier: Any .gov or .org (ACS, NIH, etc) | Top tier: Peer reviewed or RCT (NCCN, uptodate, etc.) |

Comments:

**ChatGPT:**

| Accuracy | 1 | 2 | 3 | 4 | 5 | 6 |
| --- | --- | --- | --- | --- | --- | --- |
|  | Completely incorrect | More incorrect than correct | Approximately equal incorrect and correct | More correct than incorrect | Nearly all correct | Correct |

| Completeness | 1 | 2 | 3 |
| --- | --- | --- | --- |
|  | incomplete, addresses some  aspects of the question, but significant parts are missing or incomplete | adequate, addresses all aspects of the question and provides the minimum  amount of information required to be considered complete | comprehensive, addresses all aspects of the question and provides additional information or  context beyond what was expected |

| Reference | 1 | 2 | 3 |
| --- | --- | --- | --- |
|  | Bottom tier: Any .com (webMD, etc) | Middle tier: Any .gov or .org (ACS, NIH, etc) | Top tier: Peer reviewed or RCT (NCCN, uptodate, etc.) |

Comments:

Q17: What is the recommended treatment for stage IC1 ovarian cancer?

**Google:**

| Accuracy | 1 | 2 | 3 | 4 | 5 | 6 |
| --- | --- | --- | --- | --- | --- | --- |
|  | Completely incorrect | More incorrect than correct | Approximately equal incorrect and correct | More correct than incorrect | Nearly all correct | Correct |

| Completeness | 1 | 2 | 3 |
| --- | --- | --- | --- |
|  | incomplete, addresses some  aspects of the question, but significant parts are missing or incomplete | adequate, addresses all aspects of the question and provides the minimum  amount of information required to be considered complete | comprehensive, addresses all aspects of the question and provides additional information or  context beyond what was expected |

| Reference | 1 | 2 | 3 |
| --- | --- | --- | --- |
|  | Bottom tier: Any .com (webMD, etc) | Middle tier: Any .gov or .org (ACS, NIH, etc) | Top tier: Peer reviewed or RCT (NCCN, uptodate, etc.) |

Comments:

**ChatGPT:**

| Accuracy | 1 | 2 | 3 | 4 | 5 | 6 |
| --- | --- | --- | --- | --- | --- | --- |
|  | Completely incorrect | More incorrect than correct | Approximately equal incorrect and correct | More correct than incorrect | Nearly all correct | Correct |

| Completeness | 1 | 2 | 3 |
| --- | --- | --- | --- |
|  | incomplete, addresses some  aspects of the question, but significant parts are missing or incomplete | adequate, addresses all aspects of the question and provides the minimum  amount of information required to be considered complete | comprehensive, addresses all aspects of the question and provides additional information or  context beyond what was expected |

| Reference | 1 | 2 | 3 |
| --- | --- | --- | --- |
|  | Bottom tier: Any .com (webMD, etc) | Middle tier: Any .gov or .org (ACS, NIH, etc) | Top tier: Peer reviewed or RCT (NCCN, uptodate, etc.) |

Comments:

Q18: How effective is first line treatment for stage IC1 ovarian cancer?

**Google:**

| Accuracy | 1 | 2 | 3 | 4 | 5 | 6 |
| --- | --- | --- | --- | --- | --- | --- |
|  | Completely incorrect | More incorrect than correct | Approximately equal incorrect and correct | More correct than incorrect | Nearly all correct | Correct |

| Completeness | 1 | 2 | 3 |
| --- | --- | --- | --- |
|  | incomplete, addresses some  aspects of the question, but significant parts are missing or incomplete | adequate, addresses all aspects of the question and provides the minimum  amount of information required to be considered complete | comprehensive, addresses all aspects of the question and provides additional information or  context beyond what was expected |

| Reference | 1 | 2 | 3 |
| --- | --- | --- | --- |
|  | Bottom tier: Any .com (webMD, etc) | Middle tier: Any .gov or .org (ACS, NIH, etc) | Top tier: Peer reviewed or RCT (NCCN, uptodate, etc.) |

Comments:

**ChatGPT:**

| Accuracy | 1 | 2 | 3 | 4 | 5 | 6 |
| --- | --- | --- | --- | --- | --- | --- |
|  | Completely incorrect | More incorrect than correct | Approximately equal incorrect and correct | More correct than incorrect | Nearly all correct | Correct |

| Completeness | 1 | 2 | 3 |
| --- | --- | --- | --- |
|  | incomplete, addresses some  aspects of the question, but significant parts are missing or incomplete | adequate, addresses all aspects of the question and provides the minimum  amount of information required to be considered complete | comprehensive, addresses all aspects of the question and provides additional information or  context beyond what was expected |

| Reference | 1 | 2 | 3 |
| --- | --- | --- | --- |
|  | Bottom tier: Any .com (webMD, etc) | Middle tier: Any .gov or .org (ACS, NIH, etc) | Top tier: Peer reviewed or RCT (NCCN, uptodate, etc.) |

Comments:

**Case 6: Stage IVB ovarian cancer**

Q19: How long will I live with stage IVB ovarian cancer?

**Google:**

| Accuracy | 1 | 2 | 3 | 4 | 5 | 6 |
| --- | --- | --- | --- | --- | --- | --- |
|  | Completely incorrect | More incorrect than correct | Approximately equal incorrect and correct | More correct than incorrect | Nearly all correct | Correct |

| Completeness | 1 | 2 | 3 |
| --- | --- | --- | --- |
|  | incomplete, addresses some  aspects of the question, but significant parts are missing or incomplete | adequate, addresses all aspects of the question and provides the minimum  amount of information required to be considered complete | comprehensive, addresses all aspects of the question and provides additional information or  context beyond what was expected |

| Reference | 1 | 2 | 3 |
| --- | --- | --- | --- |
|  | Bottom tier: Any .com (webMD, etc) | Middle tier: Any .gov or .org (ACS, NIH, etc) | Top tier: Peer reviewed or RCT (NCCN, uptodate, etc.) |

Comments:

**ChatGPT:**

| Accuracy | 1 | 2 | 3 | 4 | 5 | 6 |
| --- | --- | --- | --- | --- | --- | --- |
|  | Completely incorrect | More incorrect than correct | Approximately equal incorrect and correct | More correct than incorrect | Nearly all correct | Correct |

| Completeness | 1 | 2 | 3 |
| --- | --- | --- | --- |
|  | incomplete, addresses some  aspects of the question, but significant parts are missing or incomplete | adequate, addresses all aspects of the question and provides the minimum  amount of information required to be considered complete | comprehensive, addresses all aspects of the question and provides additional information or  context beyond what was expected |

| Reference | 1 | 2 | 3 |
| --- | --- | --- | --- |
|  | Bottom tier: Any .com (webMD, etc) | Middle tier: Any .gov or .org (ACS, NIH, etc) | Top tier: Peer reviewed or RCT (NCCN, uptodate, etc.) |

Comments:

Q20: What is the recommended treatment for stage IVB ovarian cancer?

**Google:**

| Accuracy | 1 | 2 | 3 | 4 | 5 | 6 |
| --- | --- | --- | --- | --- | --- | --- |
|  | Completely incorrect | More incorrect than correct | Approximately equal incorrect and correct | More correct than incorrect | Nearly all correct | Correct |

| Completeness | 1 | 2 | 3 |
| --- | --- | --- | --- |
|  | incomplete, addresses some  aspects of the question, but significant parts are missing or incomplete | adequate, addresses all aspects of the question and provides the minimum  amount of information required to be considered complete | comprehensive, addresses all aspects of the question and provides additional information or  context beyond what was expected |

| Reference | 1 | 2 | 3 |
| --- | --- | --- | --- |
|  | Bottom tier: Any .com (webMD, etc) | Middle tier: Any .gov or .org (ACS, NIH, etc) | Top tier: Peer reviewed or RCT (NCCN, uptodate, etc.) |

Comments:

**ChatGPT:**

| Accuracy | 1 | 2 | 3 | 4 | 5 | 6 |
| --- | --- | --- | --- | --- | --- | --- |
|  | Completely incorrect | More incorrect than correct | Approximately equal incorrect and correct | More correct than incorrect | Nearly all correct | Correct |

| Completeness | 1 | 2 | 3 |
| --- | --- | --- | --- |
|  | incomplete, addresses some  aspects of the question, but significant parts are missing or incomplete | adequate, addresses all aspects of the question and provides the minimum  amount of information required to be considered complete | comprehensive, addresses all aspects of the question and provides additional information or  context beyond what was expected |

| Reference | 1 | 2 | 3 |
| --- | --- | --- | --- |
|  | Bottom tier: Any .com (webMD, etc) | Middle tier: Any .gov or .org (ACS, NIH, etc) | Top tier: Peer reviewed or RCT (NCCN, uptodate, etc.) |

Comments:

Q21: How effective is first line treatment for stage IVB ovarian cancer?

Google:

| Accuracy | 1 | 2 | 3 | 4 | 5 | 6 |
| --- | --- | --- | --- | --- | --- | --- |
|  | Completely incorrect | More incorrect than correct | Approximately equal incorrect and correct | More correct than incorrect | Nearly all correct | Correct |

| Completeness | 1 | 2 | 3 |
| --- | --- | --- | --- |
|  | incomplete, addresses some  aspects of the question, but significant parts are missing or incomplete | adequate, addresses all aspects of the question and provides the minimum  amount of information required to be considered complete | comprehensive, addresses all aspects of the question and provides additional information or  context beyond what was expected |

| Reference | 1 | 2 | 3 |
| --- | --- | --- | --- |
|  | Bottom tier: Any .com (webMD, etc) | Middle tier: Any .gov or .org (ACS, NIH, etc) | Top tier: Peer reviewed or RCT (NCCN, uptodate, etc.) |

Comments:

**ChatGPT:**

| Accuracy | 1 | 2 | 3 | 4 | 5 | 6 |
| --- | --- | --- | --- | --- | --- | --- |
|  | Completely incorrect | More incorrect than correct | Approximately equal incorrect and correct | More correct than incorrect | Nearly all correct | Correct |

| Completeness | 1 | 2 | 3 |
| --- | --- | --- | --- |
|  | incomplete, addresses some  aspects of the question, but significant parts are missing or incomplete | adequate, addresses all aspects of the question and provides the minimum  amount of information required to be considered complete | comprehensive, addresses all aspects of the question and provides additional information or  context beyond what was expected |

| Reference | 1 | 2 | 3 |
| --- | --- | --- | --- |
|  | Bottom tier: Any .com (webMD, etc) | Middle tier: Any .gov or .org (ACS, NIH, etc) | Top tier: Peer reviewed or RCT (NCCN, uptodate, etc.) |

Comments:
